# Ag nanoparticles immobilized on new mesoporous triazine-based carbon (MTC) as green and recoverable catalyst for reduction of nitroaromatic in aqueous media

**DOI:** 10.1038/s41598-020-74232-4

**Published:** 2020-11-09

**Authors:** Narges Vahedi-Notash, Majid M. Heravi, Ali Alhampour, Pourya Mohammadi

**Affiliations:** Department of Chemistry, School of Science, Alzahra University, PO Box 1993891176, Vanak, Tehran, Iran

**Keywords:** Chemistry, Nanoscience and technology

## Abstract

In this research, we reported an effective method for the synthesis of a new mesoporous triazine-based carbon (MTC) substrate and its application as the green and recoverable catalyst in the synthesis of organic compounds. The porous carbon acted as a substrate for silver active species after its surface modification by chloroacetonitrile (Ag@MTC). The Ag@MTC nanocatalyst was characterized by several techniques namely, Fourier-transform infrared spectroscopy, field emission scanning electron microscopy with energy dispersive spectroscopy, X-ray diffraction, transmission electron microscopy, Brunauer–Emmett–Teller surface area analysis, and inductively coupled plasma. The Ag@MTC catalyst was applied for the reduction of nitroaromatic compounds in aqueous media by using NaBH_4_ (reducing agent) at room temperature. This nanocatalyst can be readily recovered and recycled for at least nine runs without a notable decrease in its efficiency. Catalytic efficiency studies exhibited that Ag@MTC nanocatalyst had good activity towards reduction reactions.

## Introduction

Nitrophenol compounds are categorized by the United States Environmental Protection Agency (USEPA) as the main water contaminations^1,2^ owing to their great toxicity and stability to chemical treatments and microbial degradation. Nitro compounds as the main based materials are broadly applied in medicine, ceramics, textile, cosmetics, paper, food processing, and printing industries^3,4^. Therefore, these compounds can be found at great concentrations in their effluent and severely influence aquatic life and human health^5–9^. Consequently, based on the standards presented by the USEPA^10,11^ it is required to decrease their concentrations under 10 ppm, before being released into the media.

The common wastewater treatment techniques including chemical coagulation, adsorption, precipitation, and reverse osmosis have been utilized for the removal of nitro compounds. Nevertheless, these techniques cannot be satisfactory and efficient in the decolonization of nitro compounds owing to their difficulties such as high cost and production of dangerous by-products^12–18^. Hence, it is essential to utilize environmentally friendly and very effective techniques for the removal of nitro compounds. One of the methods in wastewater treatment that has attracted notable attention recently is the utilization of noble metal based catalyst such as Pt, Pd, Ag, and Au nanoparticles owing to their high surface area, great chemical efficiency and reactivity^19^. In this respect, a broad variety of catalytic methods has been applied for the selective reduction of nitroaromatic compounds by using low-cost metal nanoparticles and alternative hydrogen sources such as H_3_NBH_3_, NaBH_4_, NH_2_NH_2_·H_2_O, etc.^20–24^. Among the noble metal nanoparticles, silver nanoparticles have attracted significant attention as a catalyst for hydrogenation reactions (hydrogenation of azo dyes and nitroaromatics) and oxidation reactions (selective catalytic oxidation of ammonia, ethylene epoxidation, etc.)^25^. Howsoever, nanoparticles with large surface areas have an intrinsic trend to decrease their energy via agglomeration^26^. To intercept the process, the nanoparticles are frequently capped with numerous stabilizers such as surfactants^27^, ligands^28^, dendrimers^29^ and polymers^30–32^. But these compounds seriously restrict the availability of the reactant particle to the catalyst surface and so diminishing the catalytic activity. To defeat this problem, nanoparticles are stabilized on numerous solid supports this issue improves the availability of their surface, and the catalyst recovery. Using the carbon mesoporous materials are widely applied as catalytic support materials due to inexpensive, great specific surface area, simple synthesis process, good chemical and thermal stability, and tunable morphological structures^33,34^. Modifying the surface of various carbon materials such as graphite, graphene, porous carbon, carbon nanotubes, nanowires, and fullerenes can chemically create new applications by improving the properties. Several atoms such as nitrogen, oxygen, sulfur, boron, phosphorus, and halogens such as fluorine, chlorine, and iodine are used as surface modifiers of carbon materials and affect surface properties. One of the most important heteroatoms used to modify carbon surfaces is the nitrogen atom. The presence of nitrogen on carbon surfaces improves surface properties, which can be attributed to increased temperature stability, increased chemical stability, increased electrical conductivity and electronic properties adjustment, active sites on carbon substrates as well as the dispersion of metal components on carbon substrates^35–37^.

In this study, we explained the synthesis of functionalized carbon mesoporous material supported by silver nanoparticles (Ag@MTC) as a recoverable nanocatalyst and it was also investigated for its catalytic activity for the reduction of nitro aromatic compounds.

## Experimental

### Chemicals

The compounds used in the catalyst synthesis as well as in the chemical reactions i.e. chloroacetonitrile (ClCH_2_CN, 99%), d ( +)-Glucose monohydrate (C_6_H_12_O_6_·H_2_O), silver nitrate (AgNO_3_, 99%), sodium hydroxide (NaOH, 98%), sodium borohydride (NaBH_4_, 98%), were prepared from Merck, Germany, Sigma Aldrich, and Fluka chemical companies. The precursor materials and solvents were all highly purified, without the need for further purification.

### Apparatus

Scanning electron microscopy (SEM) images were acquired by a SEM microscope (Tescan VEGA3, USA). X-ray diffraction (XRD) patterns were recorded on a PW 1800 X-ray diffractometer (Philips, Netherlands) with Cu-K_α_ radiation. The IR spectra of the samples were determined using the FT-IR-8400S Spectrometer (SHIMADZU, Japan). Transmission electron microscopy images were taken using a transmission microscope (Philips CM30, 300KV). N_2_ sorption analysis was performed on a volumetric adsorption analyzer (BELSORP MINI II). The specific surface areas were acquired by using the Brunauer–Emmett–Teller (BET) method.

### Synthesis of Ag@MTC nanocatalyst

The synthesis steps of silver nanoparticle catalysts on the carbon mesoporous substrate (Ag@MTC) were described below.

First, a mixture of glucose (2.0 g) with sodium hydroxide (2.7 g) was prepared in 20 mL distilled water and stirred for 12 h in an ice bath. Then a solution of 3.0 g chloroacetonitrile in 4.0 mL of isopropanol was prepared and added to the first mixture and stirred at 65 °C for 12 h. After this time, the reaction was complete by addition ethanol (70%) to the above solution and the product was separated by centrifugation and washed three times with ethanol (70%). Finally, 5.0 mL absolute ethanol was added to the above mixture and dried for 12 h at 65 °C.

Subsequently, the cyanomethyl glucose was thoroughly mixed with zinc chloride (8:1 molar ratio) and the mixture was heated for 40 h at 400 °C. After cooling down to room temperature, the mixture was rinsed with distilled water and stirred for 24 h in hydrochloric acid solution (1 M) to remove excess zinc chloride, then the obtained black powder was washed with water, tetrahydrofuran, and acetone to remove all salts and the excess hydrochloric acid, then the product was dried in a vacuum oven at 100°C^38^.

In order to synthesize silver nanoparticles on carbon mesoporous substrate, 470 mg of the glucose mesoporous substrate was first dispersed in 100 mL of water. Next, 4.72 mL of silver nitrate solution (10 mg mL^−1^) was added to the first mixture under ultrasonication and then 15 mL of 210 mg NaBH_4_ aqueous solution was gently added to the above mixture and stirred for 12 h at room temperature. Ag@MTC nanocatalyst was separated by centrifugation and dried at 30 °C^39^.

### Catalytic reduction of nitrophenols

Catalytic reduction reactions of nitro compounds in the presence of NaBH_4_ were carried out in the aqueous solution at the intended temperature. In a typical experiment, a 3.0 mL of nitroaromatic solution (1.0 mmol) was prepared and 30 mg of Ag@MTC catalyst was dispersed in this solution under 75 °C, and this mixture was continuously stirred. Next, 5.0 mmol freshly prepared NaBH_4_ solution was added to the above mixture under stirring. The progress of the reaction was controlled by a thin layer chromatography (TLC, normal hexane–ethyl acetate as solvent). After the end of the reaction, the catalyst was collected by centrifugation and the products were purified by short column chromatography over silica gel.

## Result and discussion

### Characterization of Ag@MTC nanocomposite

#### FTIR spectral analysis

**Figure 1:**
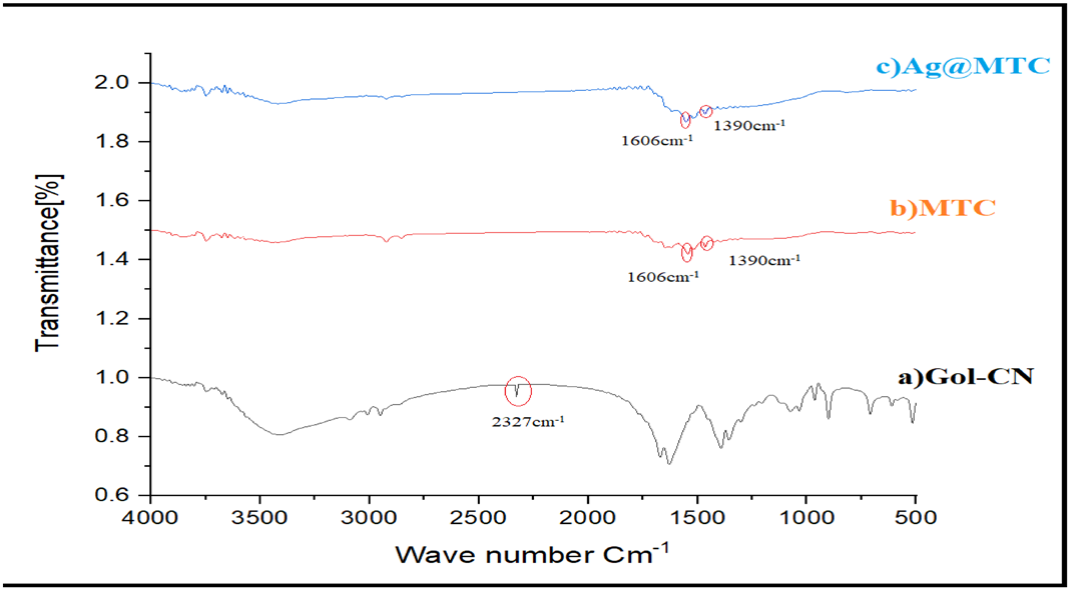
FTIR spectra of (**a**) Gol-CN, (**b**) MTC, and (**c**) Ag@MTC.

For a more detailed study of the structure of the prepared nanocatalysts, the FT-IR spectra of these materials were investigated in all three stages of (a) glucose functionalized with cyanide group (Gol-CN), (b) MTC, and (c) Ag@MTC. In the spectrum (a) of Fig. 1, the peak at the region of 2327 cm^−1^ was related to the stretching vibration of the cyanide group and this peak showed the CN groups in the glucose structure. In the spectrum (b) of MTC, the peaks observed at 1390 cm^−1^ and 1606 cm^−1^ were ascribed to the stretching vibration of C–N and C=N, respectively. In addition, the lack of the –CN group at 2327 cm^−1^ obviously indicated the successful formation of triazine rings. Finally, with the addition of silver nanoparticles to the MTC surface, there was no significant change in the spectrum (c).

#### XRD patterns of the prepared nanocatalyst

**Figure 2:**
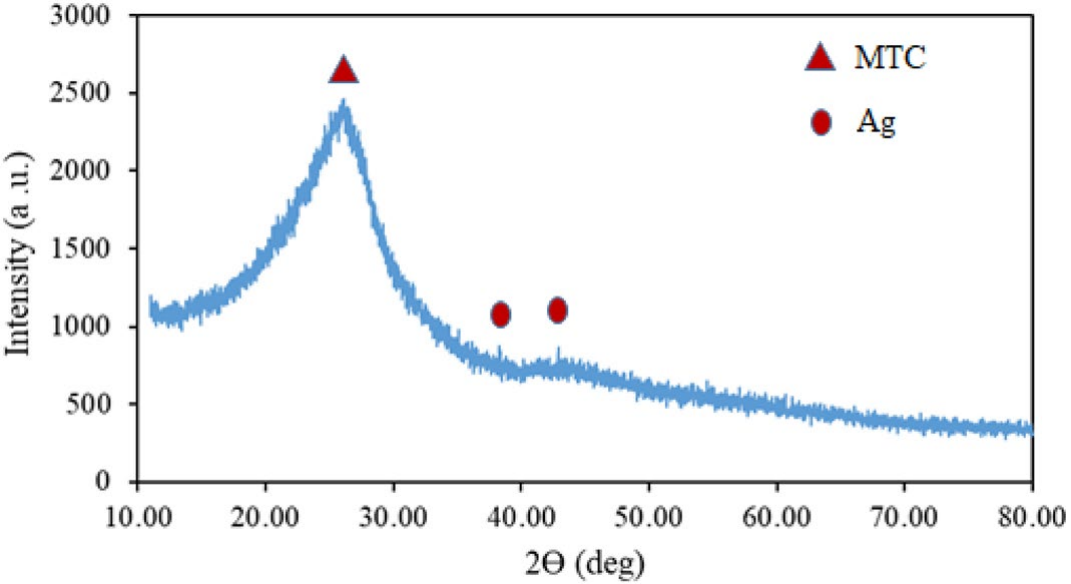
XRD pattern of Ag@MTC nanocomposite.

In amorphous materials, the X-rays are scattered in different directions, causing a large bump and are distributed over a wide range of 2Ɵ rather than narrow peaks. The XRD pattern was shown in Fig. 2. As can be seen in the spectrum, no extra peak due to another phase in the sample was observed. There are two weak peaks at 2Ɵ = 38.1 and 44.2 that related to the Ag crystals. Also, a broad peak at 2θ = 20–30 demonstrated that the sample was amorphous and had no crystalline structure, this peak related to the MCT.

#### FESEM and TEM images of Ag@MTC

**Figure 3:**
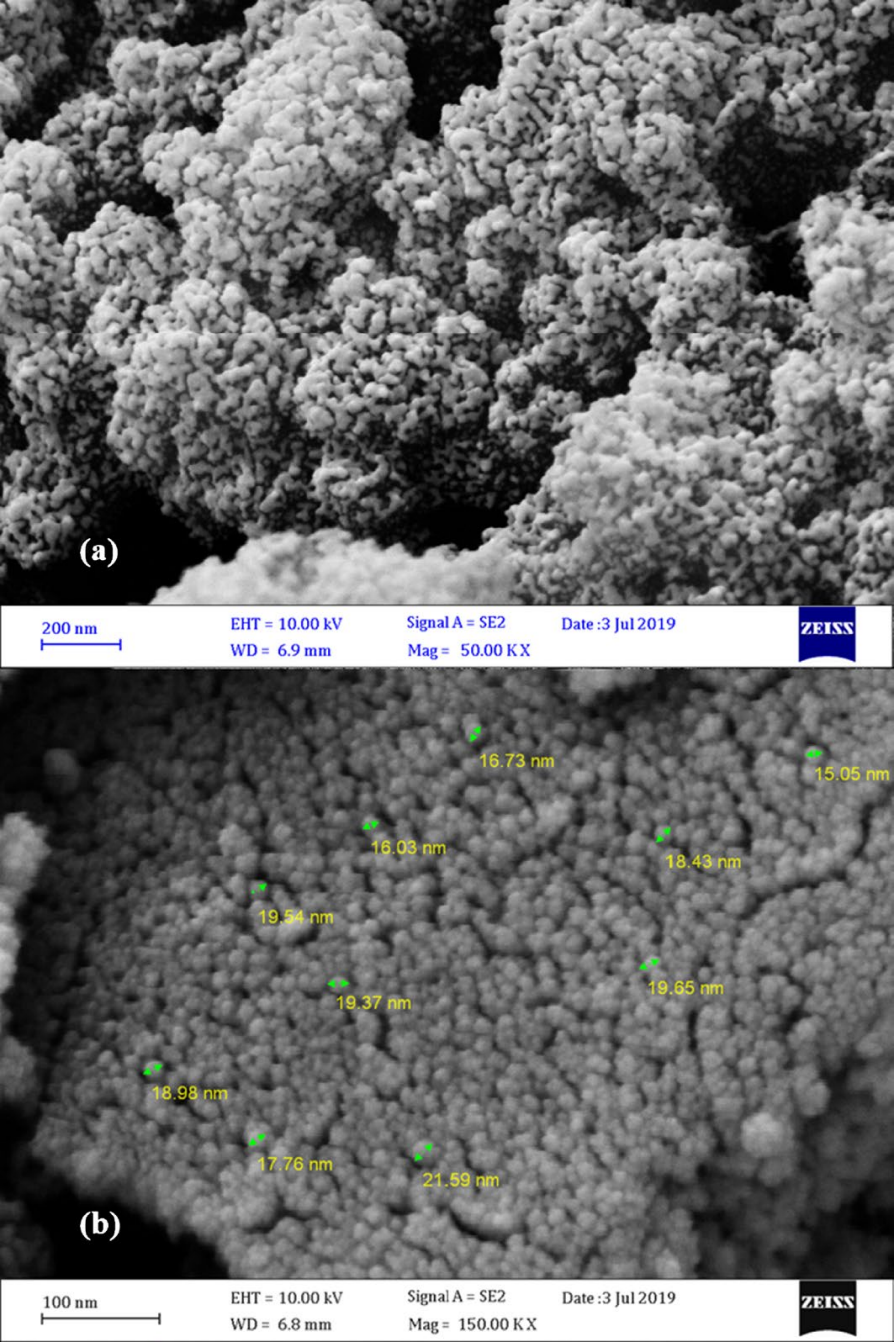
FESEM images of (**a**) MTC, (**b**) Ag@MTC.

**Figure 4:**
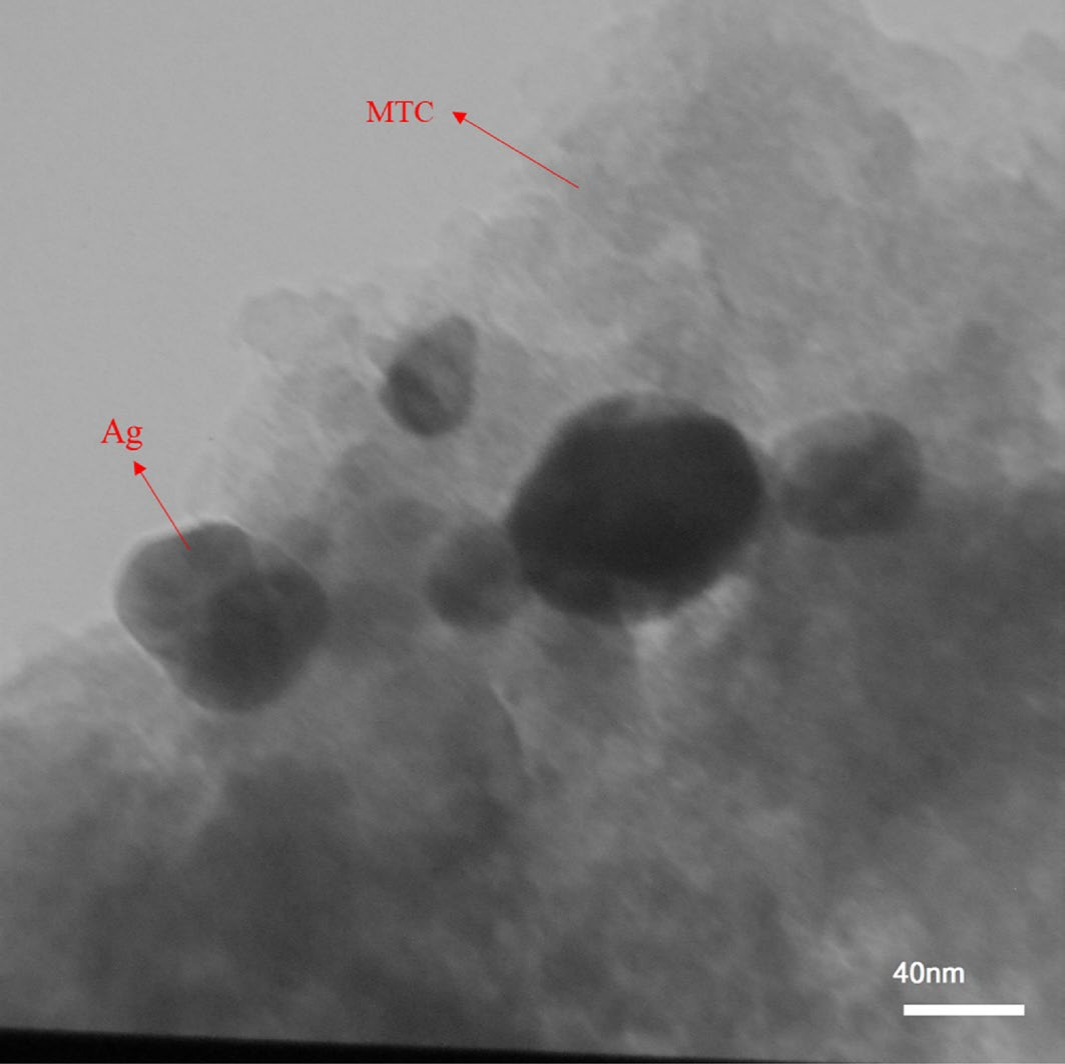
TEM of Ag@MTC.

SEM (Fig. 3) and TEM (Fig. 4) images were applied to study the texture and morphology of the prepared nanocatalyst. Figure 3 showed the porosity of the substrate and catalyst structure. The silver nanoparticles were also clearly visible in the image and were dispersed uniformly on the MTC surface and completely covered the whole surface of this support. The uniform dispersion of Ag nanoparticles on the MTC support was also approved by the TEM image. It could be observed that Ag nanoparticles had an almost spherical structure with an average diameter of approximately 16–22 nm.

**Figure 5:**
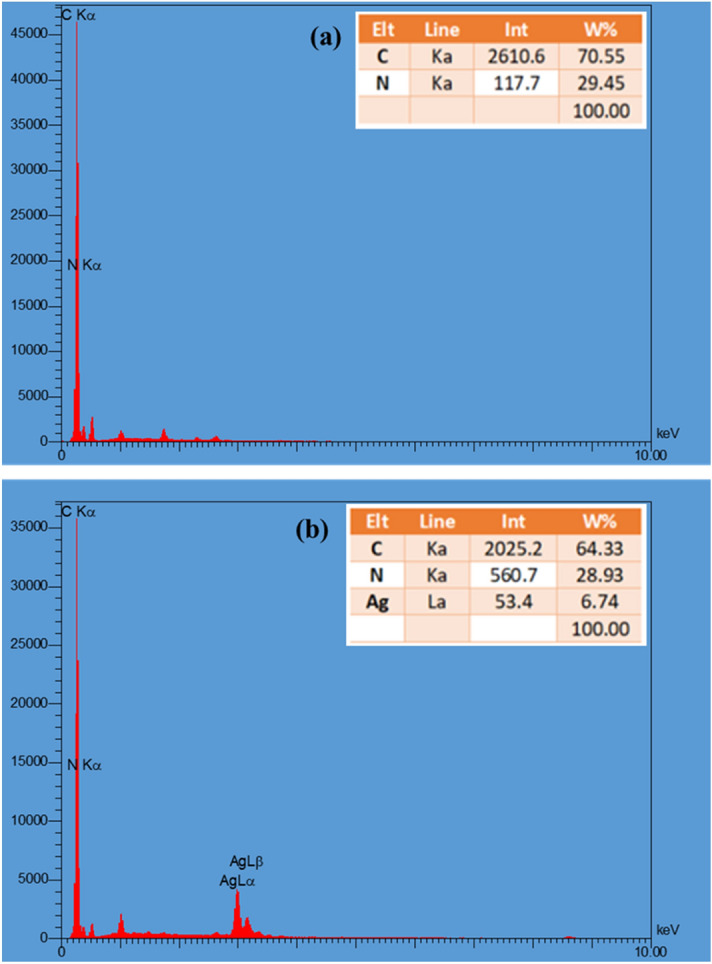
EDX analysis of (**a**) MTC, and (**b**) Ag@MTC.

**Figure 6:**
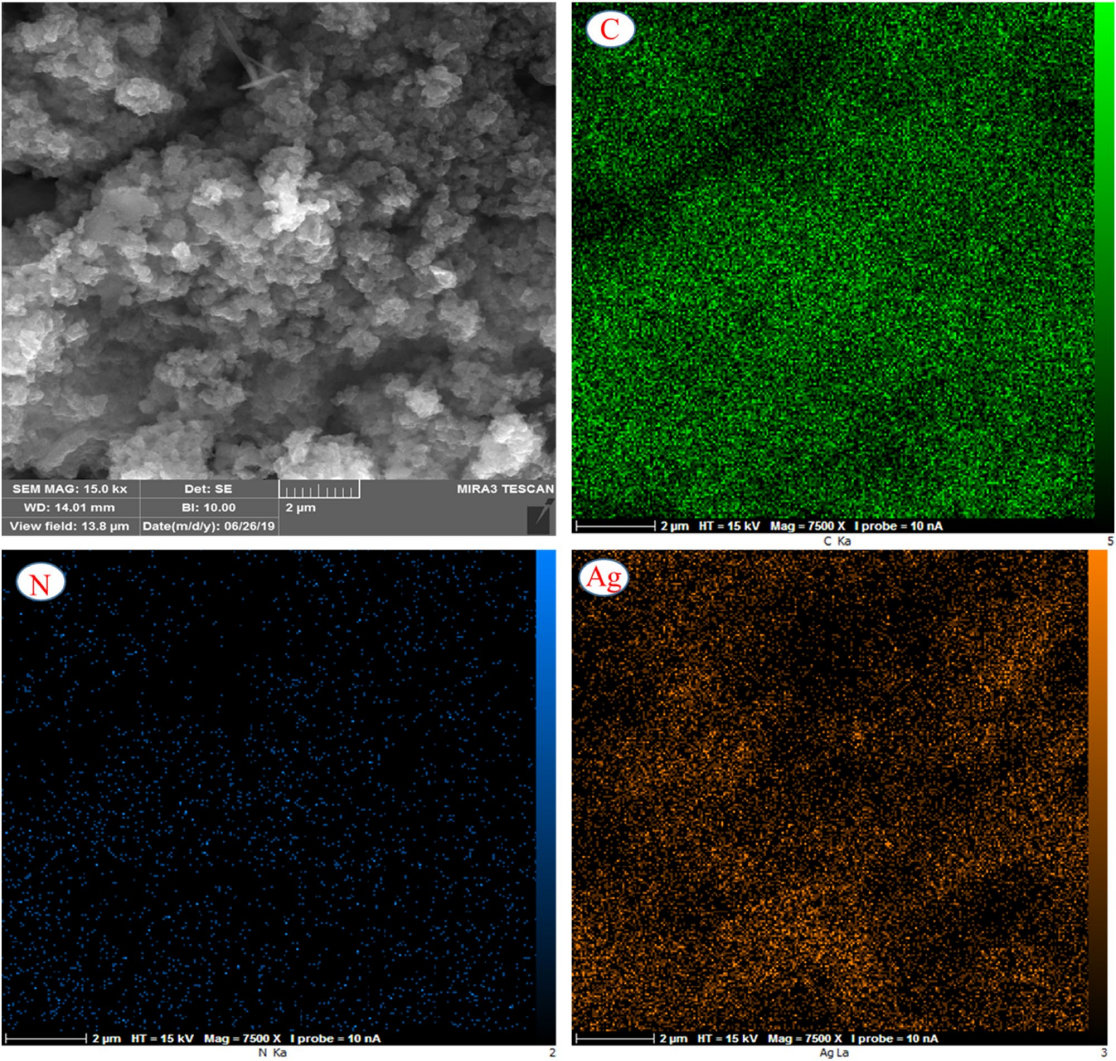
Mapping images of Ag@MTC.

Additionally, EDX analysis of the Ag@MTC nanocatalyst confirmed the existence of carbon, nitrogen, and silver elements. The weight % of C, N, and Ag was confirmed in the range of 64.33, 28.93, and 6.74, respectively, as corresponded with ICP result. The mapping tests indicated that these elements in the nanocomposite were dispersed uniformly (Figs. 5, 6).

#### N_2_ absorption–desorption study

The N_2_ absorption–desorption isotherm of the Ag@MTC nanocatalyst was given in Fig. S1. The surface area of MTC and Ag@MTC samples were obtained about 170.22 m^2^ g^−1^ and 117.29 m^2^ g^−1^, respectively. The BJH pore volume of the MTC and Ag@MTC samples were about 0.37 and 0.12 cm^3^ g^−1^, respectively, and this decrease in pore volume was due to the deposition of Ag nanoparticles on the MTC surface. In addition, the BJH pore sizes of MTC and Ag@MTC samples were about 11.76 nm and 9.19 nm, respectively.

#### ICP study

ICP analysis was used to measuring the content of silver metal present in the catalytic sample. According to the results of ICP analysis in Table 1, the content of silver loaded on the substrate was 6.7%. This was consistent with the results of the EDX analysis.

**Table 1:** ICP result of Ag@MTC.

Sample	Ag content (wt%)
Ag@MTC	6.7

#### Optimization of nitro reduction reaction conditions

To optimize the reaction conditions of nitro compounds using the Ag@MTC catalyst, the reduction reaction of 4-nitro aniline (4-NA) was considered as a model reaction. Therefore, the amount of nanocatalyst, the type of solvent, the type of reduction agent, and temperature were optimized.

#### Effect of catalyst amount on the reduction of 4-NA

In this catalysis process, the yield of reaction generally increases with increasing the amount of the Ag@MTC nanocatalyst. The yield of the reaction was considered by varying the catalyst amount from 0 to 40 mg while the other factors were kept constant. The results were summarized in Table S1. According to the results, the reduction reaction did not occur in the absence of the Ag@MTC nanocatalyst after 180 min. Using the smaller amounts of catalysts of 10 and 20 mg, the reduction reaction yields of 4-NA were found to be 100% after 90 and 70 min respectively. With the increasing catalyst amount (30 mg), the yield of the reaction was obtained 100% after 20 min. Therefore, 30 mg was determined as the optimal amount of nanocatalyst. The reaction in the presence of the catalyst alone was tested and no progress observed.

#### The effect of solvents

After investigating the appropriate amount of catalyst, the next step in optimizing the reaction conditions is to investigate the effect of the solvent in the reaction progress. Hence, the effect of several solvents on the reduction reaction was presented in Table S2. As can be understood from these results, water was the best solvent for this reaction. Reaction with water/ethanol (1: 1) solvent had a good yield but the choice of water was more favorable in terms of green chemistry. On the other hand, the ethanol solvent did not have a good yield, so the reaction with the water solvent was selected at 75 °C.

#### Effect of NaBH_4_ concentration for reduction of 4-NA

The NaBH_4_ was used as a reducing agent, this reductant cannot alone the nitro compounds reduce. So needs that the catalyst was applied. The yields of the reduction reaction were obtained at a varying concentration of NaBH_4_, retaining the other parameters constant. It was found that with enhancing NaBH_4_ concentration, the yields were increased and after a determined concentration of NaBH_4_ (5 mmol), the yields were obtained 100% after 20 min. According to the results shown in Table S3, the optimal amount for the NaBH_4_ was 5 mmol. The reaction in the presence of NaBH_4_ alone was tested and no progress observed.

#### Effect of temperature on the reduction of 4-NA

The reaction temperature represented a very significant role in the reduction of 4-NA. The reaction temperatures were varied from 65 to 95 °C while maintaining the other factors constant. The results were given in Table S4. An upsurge in reaction temperature led to obtain the higher yield. This table showed that the optimum temperature for this reaction was 75 °C.

#### Synthesis of nitroaromatic derivatives

The selectivity of a catalytic method possessed an essential role in identifying the conformity of a catalytic process. To investigate this scope, Ag@MTC catalyst was applied for the selective reduction of a diversity of nitroaromatic materials under the optimum conditions. The results were presented in Table 2. The efficiency of the catalyst indicated the completion of the reduction reactions in short times. Regardless of the presence of several functionalities in nitrobenzene derivatives, the nanocatalyst exhibited good yield (80–100%) and produced the amino products. It's interesting that under the optimum conditions, hydrazine, aldehyde, carboxylic acid, and keto groups were also endured. According to the nitroaromatic compounds with electron-donating substituents, the reduction reaction was desirable and performed in shorter reaction times. Therefore, the use of cost-effective, Ag@MTC nanocatalyst for the reduction of nitro compounds was effective.

**Table 2:**
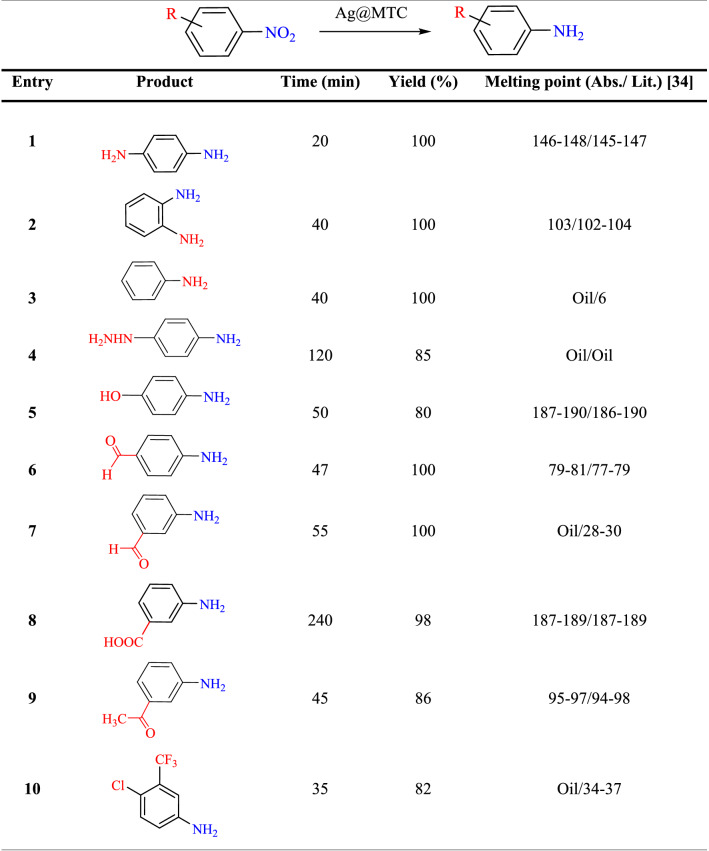
Reduction of aromatic nitro compounds in the presence of Ag@MTC.

#### Catalyst reusability

Recyclability of catalysts is an essential parameter influencing the suitable application of the catalytic system. In order to examine the recyclability of the Ag@MTC, at the completion of the reaction, the catalyst was separated by centrifuge from the reaction mixture and reutilized in the subsequent run. The obtained results revealed that the synthesized nanocatalyst could be applied at least 6 consecutive runs without a notable reduction in catalytic performance (Fig. S2). (Yield 100 to 90% and Time 15 to 16 min).

## Conclusions

In summary, we described a facile and effective process for the synthesis of Ag@MTC nanocatalyst. The prepared Ag@MTC demonstrated good catalytic performance for the reduction of nitroaromatic compounds and possessed desirable reusability. This study hence not only represented a practical method for facile preparation of Ag@MTC but also this nanocatalyst was successfully applied for the reduction of nitro compounds. The advantages of this catalyst include low cost, short reaction time, low reaction temperature, recyclability without reduced catalytic activity and non-toxicity.

## Supplementary information

Supplementary Information.
