# Supplementary material for: Ag nanoparticles immobilized on new mesoporous triazine-based carbon (MTC) as green and recoverable catalyst for reduction of nitroaromatic in aqueous media
Source: Sci Rep. 2020 Nov 9;10:19322. doi: 10.1038/s41598-020-74232-4 (PMC7653909; doi:10.1038/s41598-020-74232-4)
Supplement: Supplementary file 1 — Supplementary Information. [file 41598_2020_74232_MOESM1_ESM.docx]

**Supporting information**

**Ag nanoparticles immobilized on new mesoporous triazine-based carbon (MTC) as a green and recoverable catalyst for reduction of nitroaromatic in aqueous media**

**Narges Vahedi-Notash, Majid M Heravi *, Ali Alhampour, Pourya Mohammadi**

*Department of Chemistry, School of Science, Alzahra University, POBox 1993891176, Vanak, Tehran, Iran Tel.: +98 21 88044051; Fax: +98 21 88041344; e-mail: mmh1331@yahoo.com, mmheravi@alzahra.ac.ir*

**Figure S1.** The N_2_ absorption-desorption isotherm of the Ag@MTC nanocatalyst


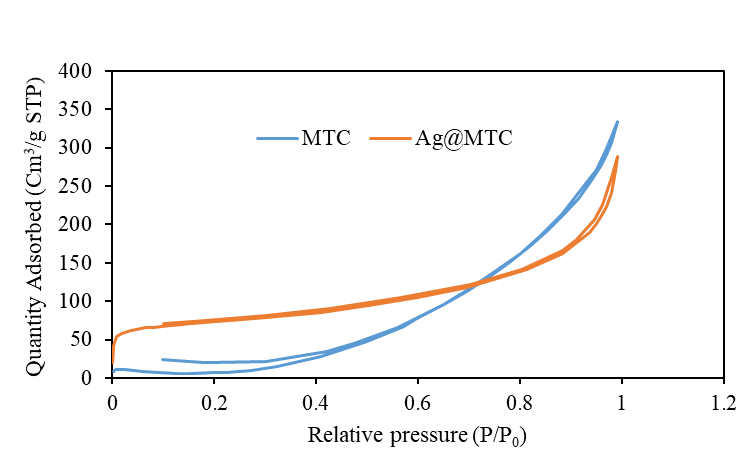


**Figure S2.** Reusability test of the Ag@MTC for model reaction


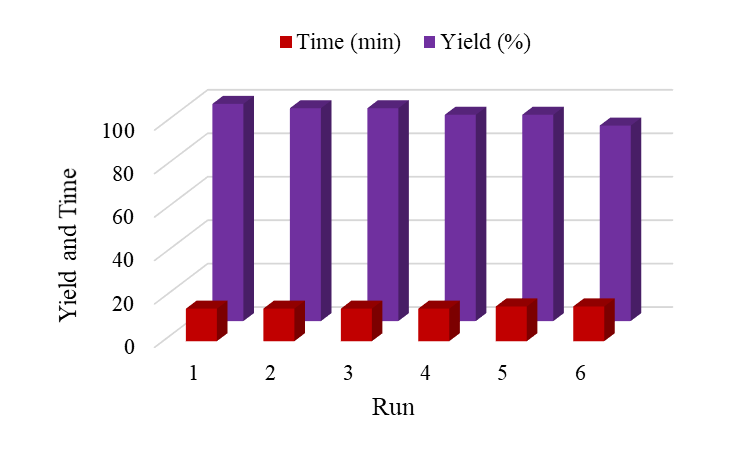


**Table S1.** Effect of catalyst amount

| **Entry** | **Catalyst amount (mg)** | | **Time (min)** | **Yield (%)** | **Selectivity (%)** |
| --- | --- | --- | --- | --- | --- |
| 1 | | 0 | 180 | 0 | 0 |
| 2 | | 10 | 60 | 100 | 100 |
| 3 | | 20 | 40 | 100 | 100 |
| 4 | | 30 | 15 | 100 | 100 |
| 5 | | 40 | 15 | 100 | 100 |

**Table S2.** Effect of solvents

| **Entry** | **Solvent** | **Time (min)** | **Yield (%)** | **Selectivity (%)** |
| --- | --- | --- | --- | --- |
| 1 | water | 15 | 100 | 100 |
| 2 | water/ethanol (1:1) | 15 | 100 | 100 |
| 3 | ethanol | 60 | 73 | 100 |

**Table S3.** Effect of NaBH_4_ concentration

| **Entry** | **NaBH_4_ concentration (mmol)** | | **Time (min)** | **Yield (%)** | **Selectivity (%)** |
| --- | --- | --- | --- | --- | --- |
| 1 | 6 | 25 | | 100 | 100 |
| 2 | 5 | 15 | | 100 | 100 |
| 3 | 4 | 40 | | 99 | 100 |
| 4 | 3 | 50 | | 99 | 100 |

**Table S4.** Effect of temperature on the reduction of 4-NA

| **entry** | **Temperature (°C)** | **Time (min)** | **Yield (%)** | **Selectivity (%)** |
| --- | --- | --- | --- | --- |
| 1 | 95 | 20 | 100 | 100 |
| 2 | 75 | 15 | 100 | 100 |
| 3 | 65 | 45 | 100 | 100 |
